# A Truth-Oriented Trust Evaluation Model of Shared Traffic Messages in the Internet of Vehicles

**DOI:** 10.3390/e27111113

**Published:** 2025-10-28

**Authors:** Jiamin Zhang, Lisha Shuai, Jiuling Dong, Gaoya Dong, Xiaolong Yang, Keping Long

**Affiliations:** 1 School of Computer and Communication Engineering, University of Science and Technology Beijing, Beijing 100083, China; zhangjm_ustb@163.com (J.Z.); promethusls@outlook.com (L.S.); hndongjiuling@163.com (J.D.); gaoyadong@ustb.edu.cn (G.D.); longkeping@ustb.edu.cn (K.L.); 2 Shunde Innovation School, University of Science and Technology Beijing, Foshan 528300, China

**Keywords:** internet of vehicles, trustworthiness of the message, trust evaluation model, smoothing factor, link analysis technology

## Abstract

The Internet of Vehicles (IoV) provides an effective solution for alleviating traffic congestion and enhancing road safety. However, shared traffic messages in IoV may deviate from on-road conditions due to self-interest protection or insufficient sensor performance. Therefore, evaluating the trustworthiness of shared messages is essential for vehicles to make informed decisions. To this end, a truth-oriented trust model for shared traffic message is proposed, which is inspired by human trust establishment mechanisms (HS-TEMs). Firstly, we quantify the integrated trust value (I-VT) of the message sender by fusing self-experience-based vehicle trust (SEB-VT) and peer-recommendation-based vehicle trust (PRB-VT). In SEB-VT, a sample-size-dependent smoothing factor dynamically trades off prior information and empirical evidence, reducing instability under small-sample conditions. In PRB-VT, we employ link analysis to compute the reference degree of recommendation information, which mitigates biases arising from subjective cognitive limitations. Secondly, with the I-VT of vehicles, we calculate event trust (ET) by differentiating message attitudes and quantifying their relative influence, which effectively reduces the impact of individual bias on the final judgment. The simulation results show that HS-TEM can accurately and fairly evaluate the credibility of messages, which helps vehicles make informed decisions.

## 1. Introduction

The Internet of Vehicles (IoV) is an effective means to improve urban traffic problems. In recent years, traffic accidents and road congestion have occurred frequently and are on the rise, which has seriously affected people’s travel efficiency and the safety of life and property [1]. Solving urban traffic problems is a complex system engineering. In addition to relying on the technological innovation of vehicles themselves, it also requires the coordinated cooperation between people, vehicles and roads [2]. IoV technology, the application of the Internet of Things in the field of transportation, enables vehicles (referring to those on the road) to share traffic-related messages with various traffic elements [3]. These shared messages help vehicles gain a broader field of vision and more time to respond to emergencies, and helps to realize functions such as collision warning and dynamic path planning [4].

**Figure 1:**
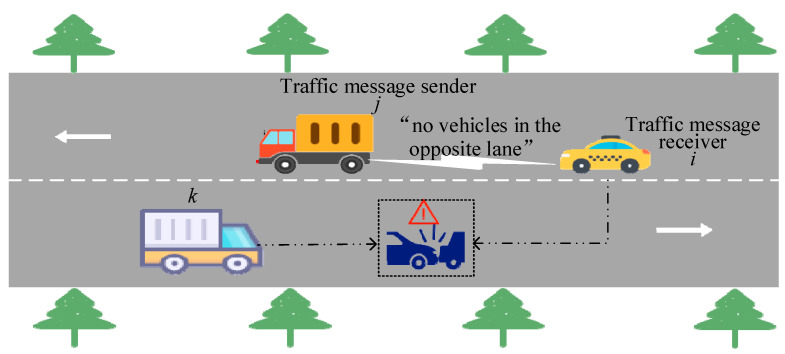
Schematic diagram of overtaking risk in opposing lanes based on false information.

However, the trustworthiness of traffic messages shared within the IoV remains to be evaluation. Due to factors such as self-interest protection, limitations in sensor performance, and heterogeneous observational perspectives [5], the shared traffic message may deviate from actual on-road conditions. If such false message is mistakenly trusted, it can hamper traffic efficiency or even pose risks to the lives and property of passengers and drivers [6]. As illustrated in Figure 1, consider a scenario in which vehicle i intends to overtake by temporarily using the opposite lane, but its own view of the oncoming traffic is obstructed. In this case, the preceding vehicle j may maliciously disseminate false message by claiming that the opposite lane is clear, even though an oncoming vehicle k is approaching. If vehicle i trusts this false message and proceeds to overtake, a collision accident may occur. Therefore, establishing an effective mechanism to evaluate the trustworthiness of shared traffic messages in the IoV constitutes an imperative task.

In response to this challenge, researchers have introduced concept of trust from sociology into the field of IoV security [7]. In the context of IoV, trust is defined as the faith that a trustor vehicle places in another trustee vehicle for sharing trusted and authentic messages [8,9]. Some researchers have proposed message-centric trust models, which make direct assessments regarding the trustworthiness of shared traffic message content. These methods primarily involve logical analysis, fact verification, and information research applied to shared traffic message to determine its trustworthiness. For example, Gurung et al. [10] have adopted a tier-oriented and analytical methodology, where vehicles continuously evaluate the reliability of gathered message. This evaluation is contingent upon factors such as content similarity, content conflict, and routing similarity among various messages associated with a specific event. Shaikh and Alzahrani [11] proposed a message-oriented trust model, which computes a trust value for each message received from multiple sources, clusters the messages, and then considers the category of messages with a high trust value as a trusted message. Rehman et al. [12] introduced a context-aware trust management framework for the IoV, wherein the framework learns the environment from received reports, establishes context around events, and identifies false messages by detecting anomalies. Mirzadeh et al. [13] employed subjective logic operations on information fragments collected from other connected vehicles to filter out malicious messages. However, most of such existing models necessitate the collection of substantial evidence related to the current event to compute the trustworthiness of shared traffic message. This approach can be time-consuming and may yield outdated results. Additionally, trust models may sometimes fail to collect sufficient evidence, resulting in inaccurate outcomes.

To address these emerging challenges, other researchers have also proposed models that assess shared traffic message trustworthiness by leveraging the trustworthiness of the message senders. This type of model is grounded in the notion that the interaction behavior of vehicles exhibits a certain degree of consistency, meaning that past behavior can serve as a reliable predictor of future behavior. Yang et al. [14] proposed a distributed trust management scheme that calculates the probability of an event based on all relevant messages received by the evaluator, subsequently evaluating the trustee and aggregating these evaluations to determine the trustee’s trustworthiness. If the trust value falls below a specified threshold, the message send by trustee is considered untrustworthy. Ge et al. [15] developed a provenance-aware trust model, which uses a hash function to identify the integrity of the message and derive node trust values based on it. Ahmad et al. [16] designed a trust model resistant to Man-in-the-Middle attacks. In this model, the direct trust value of the trustee is computed based on the trustor’s observations of their sharing and forwarding rates. Additionally, it configures confidence in recommendation information based on the geographic location between the sender and receiver. Alnasser et al. [17] presented a recommendation-based trust model to safeguard the IoV against false message, which divides confidence levels for recommended information based on the comprehensive trust value of the recommender. The aforementioned trust models have made notable progress in assessing the trustworthiness of shared traffic message. However, they have the following shortcomings: (1) the computation of the referability level of recommendation information is unduly influenced by the evaluator’s subjective perspective; (2) the model’s accuracy relies too much on the consistency of vehicle behavior and lacks applicability.

In order to overcome the aforementioned limitations and achieve an impartial trustworthy assessment of the shared traffic message, we propose a truth-oriented trust model. Our primary contributions can be summarized as follows:

(1) To effectively deal with the diversity of vehicle behaviors and objectively evaluate the vehicle’s trustworthiness, we jointly quantify the vehicle’s integrated trust value (I-VT) using both self-experience-based vehicle trust (SEB-VT) and Peer-recommendation-based vehicle trust (PRB-VT).
(2) In SEB-VT, a sample-size–dependent smoothing factor dynamically trades off prior information and empirical evidence, reducing small-sample instability and improving the accuracy and stability of trust evaluation.
(3) In PRB-VT, we leverage link analysis techniques to compute the reference degree of recommendation information, enabling impartial assessment of its quality under heterogeneous recommender reliability and sensor performance.
(4) To reliably determine the trustworthiness of shared traffic message and improve the applicability of the model, we calculate event trust (ET) by differentiating message attitudes and quantifying their relative influence, which effectively reduces the impact of individual bias on the final judgment.
(5) By incorporating entropy as a supplementary metric, we validate the soundness of the proposed smoothing mechanism. Moreover, to validate effectiveness and robustness of our proposed model, we conduct simulation studies demonstrating that the model maintains high precision and recall under diverse attack models.

The remainder of this paper is structured as follows. Section 2 introduces the system model, which provides the foundation for Section 3, where the proposed trust model is outlined. Building on this, Section 4 presents the simulation analysis of the model. Finally, Section 5 concludes with a summary of the overall work conducted.

## 2. System Model

**Figure 2:**
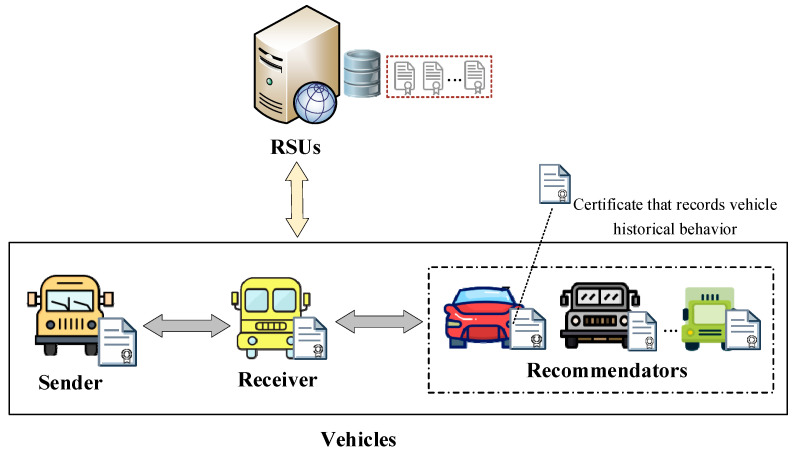
System model of truth-oriented trust evaluation in vehicular networks.

The system model of truth-oriented trust evaluation in vehicular networks is depicted in Figure 2. This model primarily includes two types of entities: vehicles acting as senders or receivers, and Roadside Units (RSUs).

(1) Vehicles (senders or receivers): Vehicles perceive road conditions through onboard sensors and share the sensed road condition messages with other vehicles via the vehicle-to-vehicle communication standards (e.g., the cellular vehicle-to-vehicle (C-V2V) [18] or dedicated short-range communications (DSRC) [19]). This decentralized message dissemination ensures low latency and local adaptability, both of which are critical for vehicular safety applications. The vehicle receiving the message requests the recommenders’ reference degrees from the RSUs and integrates multi-source evidence to evaluate the message’s trustworthiness, thereby facilitating appropriate actions. After acting on the message, the receiver generate recommendation feedbacks for recommenders based on the trustworthiness and then upload these feedbacks into the RSU. In addition, each vehicle maintains a certificate that records its historical behavior, which contains the identities of the vehicles that have interacted with it and the trust values of these vehicles.
(2) RSUs: RSUs serve as trusted infrastructure that complements vehicle-side computation by collecting, computing, and managing long-term trust evidence (e.g., uploaded recommendation feedback and the resulting reference degrees). Specifically, the RSU maintains a repository of recommendation feedback from vehicles and, based on this data, computes each recommender’s reference degree to quantify the credibility of their recommendations. RSUs then provide this information to vehicles via on-demand queries.

## 3. A Truth-Oriented Trust Evaluation Model

**Figure 3:**
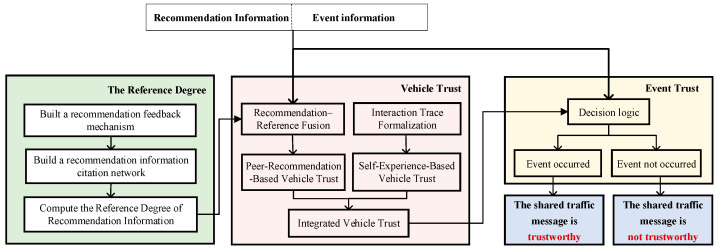
The overview of the truth-oriented trust model.

The proposed model serves the purpose of safeguarding connected vehicles against erroneous shared traffic message, thereby ensuring that IoV can effectively improve traffic safety and efficiency. To this end, we present a truth-oriented trust model to evaluate the trustworthiness of shared traffic message. This model includes three parts, i.e., the vehicle trust, the reference degree, and the event trust, as shown in Figure 3.

### 3.1. The Trustworthiness of Vehicles

#### 3.1.1. Self-Experience-Based Vehicle Trust (SEB-VT)

The self-experience-based vehicle trust represents the trustworthiness perception of trustor vehicle *i* (i.e., vehicle receiving shared traffic message) towards the trustee vehicle *j* (i.e., the vehicle sharing traffic message), which is derived from *i*’s own interaction experience with *j*. Typically, SEB-VT is measured based on the proportion of authentic road traffic message shared by trustee vehicles over historical time, and as referred to in [17,20], this approach is grounded in the idea that historical behavior is the important basis for assessing future reliability. However, calculating SEB-VT based solely on this proportion may result in significant errors when there are limited interactions among vehicles.

To address this issue, we propose a sample-size-based smoothing factor *d*(*x*) that adaptively balances between prior knowledge and empirical estimation, thereby mitigating instability under small samples. The equation of the *d*(*x*) is as follows:

(1) $$d(x)=\frac{\arctan(x-\sigma )}{\pi }+0.5$$

where *x* denotes the total number of interactions among vehicles; *σ* is an adjustment factor, and its range of values can be determined with reference to [21,22].

Assuming that in the *k*th time period, *m* represents the cumulative count of road traffic events shared by collaborative vehicle *j*, and *s* denotes the number of authentic events shared by vehicle *j*, the SEB-VT of vehicle *j* is computed as follows:

(2) $$ST_{ij}(k)=d(m)*\frac{s}{m}+(1-d(m))*A_{0}$$

where *A*_0_ represents the default SEB-VT of the collaborative vehicle, depicting the initial value when no interaction has occurred (e.g., *A*_0_ = 0.5); *d*(*m*) is the weight of the numerical value in Equation (2).

To justify the rationality of smoothing factor *d*(*m*), we analyze how the uncertainty of the self-experience-based vehicle trust (SEB-VT) *ST_ij_* changes with sample size *m* when the smoothing *d*(*m*) is applied. We use Shannon entropy to quantify and interpret this uncertainty. For each time interval *k*, we define an entropy-based measure *H_k_* and derive a corresponding certainty indicator *D_k_* from *H_k_*.

(3) $$H_{k}=-\left[\begin{array}{c} ST_{ij}(k)\log ST_{ij}(k)+(1-ST_{ij}(k))\log(1-ST_{ij}(k)) \end{array}\right]$$

(4) $$D_{k}=1-H_{k}/log2$$

**Figure 4:**
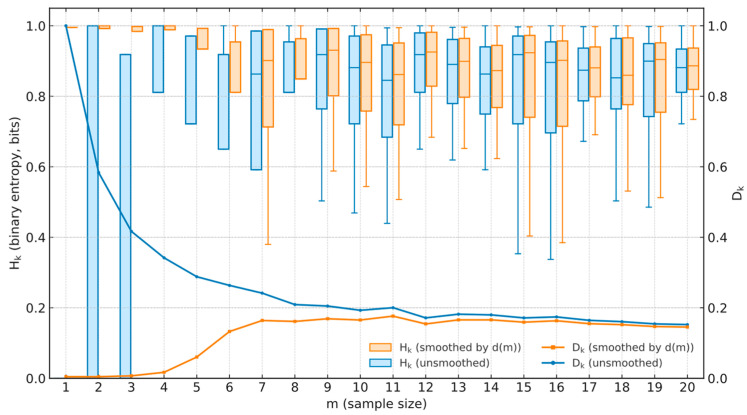
Entropy-based validation of the *d*(*m*) smoothing rationality.

The essential role of *d*(*m*) is to regulate the transition between small-sample reliance on the prior *A*_0_ and large-sample reliance on the observed ratio *s*/*m*, thereby mitigating fluctuations under limited samples and ensuring convergence of trust values as the sample size increases. Meanwhile, Figure 4 contrasts binary entropy *H_k_* (left axis, boxplots) and decisiveness *D_k_* (right axis, curves) for the unsmoothed *ST_ij_* and the *ST_ij_* smoothed by *d*(*m*). Without smoothing, small samples exhibit deceptively low entropy with large dispersion (overconfidence). With *d*(*m*), entropy is higher at small *m* (acknowledging uncertainty) and decreases and concentrates as *m* grows, while *D_k_* increases and stabilizes. These trends align uncertainty with evidence—high at small samples, low at large—thereby providing an information-theoretic validation and explanation for the necessity of *d*(*m*) in bridging prior and empirical data.

#### 3.1.2. Peer-Recommendation-Based Vehicle Trust (PRB-VT)

In practical scenarios, the trustor vehicle often lacks continuous direct interaction experience with every trustee vehicle. Even when such interactions occur, the trustor vehicle’s observations are inherently limited to a partial perspective of the trustee vehicle’s behavior. This limitation arises from the high mobility of vehicles and the dynamic, heterogeneous nature of their behaviors. Hence, in order to improve the practicality and fairness of the model, it is necessary to introduce the recommendation information of other vehicles (i.e., recommenders) for the trustee vehicle when evaluating the trust of the trustee vehicle. The specific steps for computing recommendation trust are outlined below.

(1) Acquisition of recommendation information

**Figure 5:**
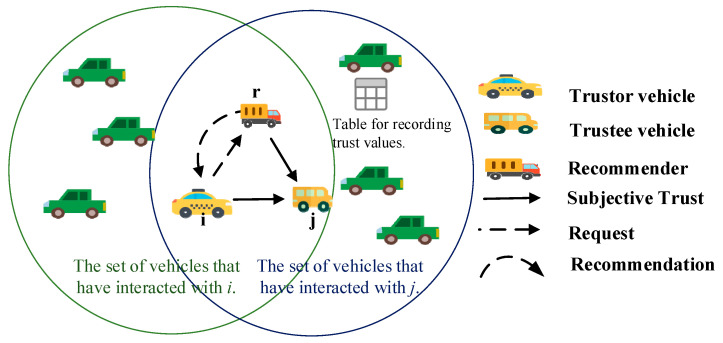
Acquisition of recommendation information.

The process for trustor vehicle *i* to obtain recommendation information about trustee vehicle *j* is as follows, as depicted in Figure 5. In VANETs, each vehicle maintains a trust table that records the subjective trust values of all vehicles it has interacted with. When vehicle *i* serves as the road traffic data receiver and vehicle *j* as the sharer of road traffic data, vehicle *i* selects neighboring vehicles that have interacted with both *i* and *j* as recommenders (e.g., *r*) based on their respective trust tables. Subsequently, vehicle *i* sends a recommendation request $RMsg=(ID_{i},ID_{r},t_{ir},RT_{rj}(k),\sigma_{i})$ via multi-hop broadcast to the selected recommenders. Among them, *ID_i_* and *ID_r_* represent the identity identifiers of vehicles *i* and *r*; *t_ir_* denotes the time at which the request is sent; ${RT}_{rj}(k)$ means the content of the request, that is, *r*’s recommendation information for *j*; *σ_i_* is the signature of vehicle *i* issuing this request. The recommender who receives the request replies to vehicle *i* with their recommendation information for vehicle *j* ${ST}_{rj}(k)$. The message in response is expressed as follows: $REMsg=(ID_{r},ID_{i},t_{ri},ST_{rj}(k),\sigma_{r})$.

(2) Computing the reference degree of recommendation information

Due to the recommender having varying levels of reliability and sensor performance, not every recommendation information carries the same reference value. Therefore, we need to compute the reference degree of the recommendation information for its reasonable utilization. In response to this requirement, we propose a method that leverages collective intelligence to compute reference degree. Specifically, we use the level of approval from the public regarding the recommender’s historical recommendation information as the reference degree for their current recommendation. This method significantly reduces the negative effects of subjective cognitive bias compared to traditional methods [16,17,20]. Unlike traditional trustor-centric weighting (e.g., Refs. [16,17,20]), which assigns a recommender’s confidence according to a single evaluator’s trust toward that recommender, our model computes a reference degree via RSU-side link analysis over the global recommendation graph. This group-derived credibility aggregates multi-receiver, multi-event evidence and is therefore less sensitive to any individual’s cognitive bias or local sensing limitations, leading to a reduced impact of subjective bias in the final trust fusion. The computation process is as follows:

First, we built a recommendation feedback mechanism that assess the reliability of previously provided recommendation information by the recommender. Specifically, we compute the deviation between the integrated trust value ${GT}_{ij}(k)$ (global view, its computation is provided in Equation (8)) and the recommendation-provided trust ${ST}_{rj}(k)$ (individual view) offered by recommender *r*. This deviation quantifies how consistent the recommender’s opinion is with the collective evaluation results, and it is defined as follows:

(5) $$dv_{ij}^{rj}(k)=|GT_{ij}(k)-ST_{rj}(k)|$$

We judge the deviation against a data-driven tolerance $\tau_{k}$. Specifically, let $R_{ij}(k)$ denote the set of recommenders who, during interval k, supplied vehicle i with recommendation information concerning trustee j. For time interval k, we construct the deviation set $D_{ij}(k)=\{d_{ij}^{rj}(k)=|GT_{ij}(k)-ST_{rj}(k)|:r\in R_{ij}(k)\},$ and define $\tau_{k}$ as the (1−α)-quantile of $D_{ij}(k)$ (default α=0.05). A recommendation information from r is regarded as reliable if $d_{ij}^{rj}(k)\leq \tau_{k}$; otherwise, it is considered unreliable.

**Figure 6:**
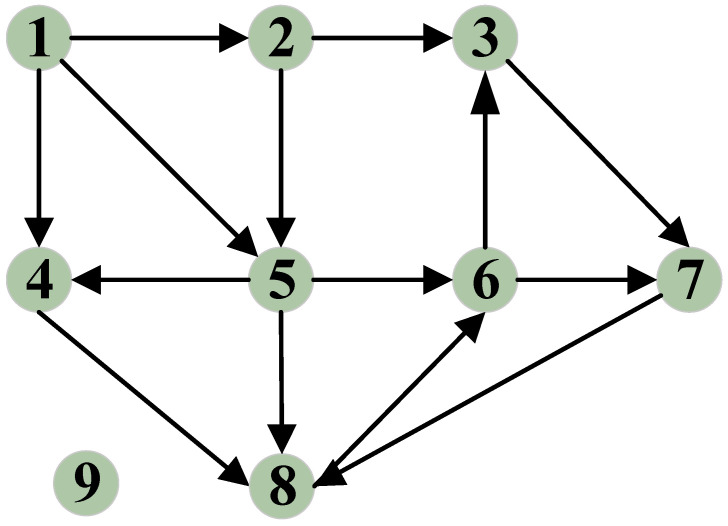
A recommendation citation network that illustrates the citation dynamics of recommendation information historically provided by recommenders.

Second, we create a recommendation citation network based on the recommendation feedback mechanism. If recommender *r* provides reliable recommendation information for vehicle *i*, a directed edge is established between *r* and *i*. We then gather all the edges formed between vehicles and the vehicles connected by these edges to construct a directed graph, which we refer to as the recommendation citation network. This network represents the references to recommendation information between vehicles and can be expressed as *G =* (*V*, *R*). Here, *V* is the set of vehicle pairs formed by vehicles citing recommendation information and the providers of this information, and *R* represents the set of directed edges. As depicted in Figure 6, a directed edge exists between nodes 6 and 7 because node 7 cites recommendation information provided by vehicle 6. Conversely, node 9 is currently isolated as its recommendation information has not been cited by other nodes.

Next, we compute the reference degree of recommendation information by exploring the recommendation citation network. The reference degree of the current recommendation information depends on the quantity and quality (i.e., trust value) of vehicles referencing the historical recommendation information provided by the recommender. A higher number and better quality of vehicles referring to a recommendation information provider indicate a more reliable provider. The reference degrees are computed as follows:

(6) $$CV_{r}(k)=c\sum_{g\in S_{r}}\frac{CV_{g}(k-1)}{n_{g}^{out}}+\frac{1-c}{Q},r=1,2,\ldots ,Q$$

Here, *r* is the provider of the recommendation information, *c* is the scaling factor used to ensure the convergence of the algorithm (Page and Brin, the originators of the PageRank algorithm, suggest *c* = 0.85), *S_r_* is the set of vehicles citing the recommendation information of *r* in the directed graph *G*; $N_{S_{r}}$ denotes the number of vehicles in $S_{r}$; $n_{g}^{out}$ is the number of recommendations cited by vehicle *g* in the *k* − 1 computation period; *Q* is the total number of nodes in the directed graph; ${CV}_{g}(0)$ is set to 1/$N_{S_{r}}$.

(3) Measurement of recommendation trust

The computation of recommendation trust involves a weighted average of recommendation information supplied by several recommenders. We use the reference degree of recommendation information obtained in Section 2 as the weight of each recommendation information. Let us assume the total number of recommenders is *p*, and then the recommendation trust is described by the formula below:

(7) $$RT_{ij}(k)=\sum_{r=1}^{p}\left(\frac{CV_{r}(k)}{\sum_{y=1}^{p}CV_{y}(k)}\times ST_{rj}(k)\right)$$

#### 3.1.3. Integrated Vehicle Trust (I-VT)

To effectively deal with the diversity of vehicle behaviors and objectively evaluate the collaborative vehicle’s trustworthiness, we jointly quantify the collaborative vehicle’s integrated trust value using both self-experience-based vehicle trust *ST_ij_* and Peer-recommendation-based vehicle trust *RT_ij_*. The comprehensive trust value of collaborative vehicle *j* is determined by the following formula:

(8) $$GT_{ij}(k)=\alpha ST_{ij}(k)+\beta RT_{ij}(k)$$

We employ a weighted fusion of self-experience-based and peer-recommendation-based trust values to compute the integrated trust value of the collaborative vehicle, thus preventing undue favoritism towards either perspective.

### 3.2. The Trustworthiness of Events

Event trust helps the connected vehicle to evaluate whether the traffic data they receive about a specific road traffic event is trustworthy, thereby achieving trusted environmental sensing. Usually, connected vehicles receive traffic data shared by several collaborative vehicles. Each piece of traffic data contains information about whether an event occurred. Therefore, trustor vehicles create two sets, a positive set and a negative set, after receiving traffic data about an event. The positive set gathers collaborative vehicles reporting the occurrence of events along with their associated trust values, while the negative set gathers collaborative vehicles reporting the non-occurrence of events along with their respective trust values. Among them, the trust value of the collaborative vehicle is obtained by Equation (8). We assume that trustor vehicle *i* has *M* collaborators in the positive set and *N* collaborators in the negative set. The average trust of sets is determined by

(9) $$T_{k}^{Positive}(i)=\frac{\sum_{a\in M}GT_{ia}(k)}{\left|M\right|};T_{k}^{Negative}(i)=\frac{\sum_{b\in N}GT_{ib}(k)}{\left|N\right|}.$$

Considering vehicles possess different historical trust levels, which reflect their sensing accuracy and behavioral credibility. Using a simple arithmetic mean, as in Equation (9), assumes that all collaborators contribute equally, which may lead to biased event trust when low-trust vehicles provide erroneous or malicious reports. To mitigate this, we introduce a trust-proportional weighting scheme, where each collaborator’s contribution is scaled according to its individual trust value $GT_{ia}(k)$ or $GT_{ib}(k)$. This approach allows highly trusted vehicles to exert greater influence on the aggregated event trust, while reducing the impact of low-trust or potentially unreliable participants. To this end, the weight of the *a*-th collaborator in the positive set and the weight of the *b*-th collaborator in the negative set are computed according to

(10) $$\omega_{ia}(k)=\frac{GT_{ia}(k)}{T_{k}^{Positive}(i)};\;\omega_{ib}(k)=\frac{GT_{ib}(k)}{T_{k}^{Negative}(i)}$$

Event trust is the weighted average of road traffic message in the set, and the event trust for positive and negative sets is calculated by

(11) $$Tavg_{k}^{Positive}(i)=\frac{\sum_{a\in M}\omega_{ia}(k)*GT_{ia}(k)}{\left|M\right|};\;Tavg_{k}^{Negative}(i)=\frac{\sum_{b\in N}\omega_{ib}(k)*GT_{ib}(k)}{\left|N\right|}$$

If the subsequent decision rule is satisfied, the event is deemed to have taken place.

(12) $$Tavg_{k}^{Positive}(i)-Tavg_{k}^{Negative}(i)\geq 0$$

Then, the traffic message in the positive set is considered trustworthy. Otherwise, it is considered non-referential.

(13) $$De\mathrm{cision}=\left\{\begin{array}{ll} Trusted, & Tavg_{k}^{Positive}(i)-Tavg_{k}^{Negative}(i)\geq 0\\ Non-referential, & otherwise \end{array}\right.$$

## 4. Experimental Results and Analysis

### 4.1. Simulation Settings

**Figure 7:**
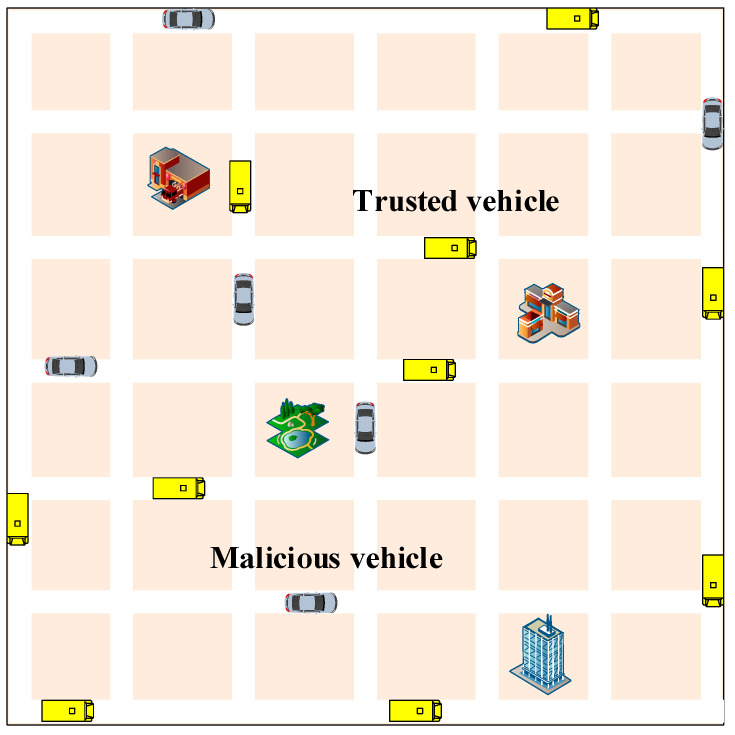
The road traffic model.

In our simulation, we utilize the road traffic model depicted in Figure 7 as the simulation environment. It consists of fourteen crisscrossing roads. Specific simulation parameters are detailed in Table 1. To create a more realistic representation of autonomous vehicle traffic scenarios, we introduce traffic events and vehicles randomly into the simulation. The vehicle movement model is based on a stochastic model (e.g., city section mobility model), with vehicles continuously broadcasting Cooperative Awareness Messages (CAM) to enable other vehicles to gather information about their movements. In the event of a traffic incident, the vehicle transmits Decentralized Environmental Notification Messages (DENM) to inform other vehicles of the current traffic situation. The yellow vehicles represent regular vehicles, while the blue vehicles indicate malicious vehicles randomly chosen from the initialized vehicle pool. For the urban-area scenario with randomly deployed vehicle positions and traffic events, we generated 300 independent instances (different random seeds), ran our model and the baselines (i.e., the recommendation-based model proposed in [17]) to compute precision and recall, and report the average results.

To validate the effectiveness and robustness of the proposed model, we establish two typical scenarios and an attack model, as outlined in Table 2. Scenario 1 is a naive fake traffic message attack scenario, where malicious vehicles deliberately share message that deviates from actual road conditions on its own. Scenario 2 is a sly fake traffic message attack scenario. In this scenario, malicious vehicles collude with other vehicles to collectively share false traffic message that contradict actual road conditions. Furthermore, the attack model is a non-friendly recommendation attack, which takes into account malicious vehicles that tout or denigrate collaborative vehicles.

### 4.2. Performance Evaluation Metrics

Since the fundamental purpose of the trust model is to identify the reliability of message traffic message and thereby prompt AVs to make correct decisions, we intend to evaluate the solution’s performance from the accuracy perspective. References [23,24], we employ precision and recall as indicators to evaluate the model’s performance.

- Precision—It depicts the ability of the trust model to correctly identify false message traffic message. The formula for precision is defined as follows:

(14) $$Precision=\frac{N_{F|F}}{N_{F|H}+N_{F|F}}$$

where *N_F|H_* represents the number of trusted messages misjudged as false messages and *N_F|F_* represents the number of false messages correctly identified by the trust model.

- Recall—It describes the trust model’s detection rate on false traffic message. Then recall can be expressed as:

(15) $$Recall=\frac{N_{F|F}}{N_{H|F}+N_{F|F}}$$

where *N_H|__F_* represents the number of messages that the trust model mistakenly determines false messages as trustworthy.

### 4.3. Simulation Results and Analysis

#### 4.3.1. The Performance of Proposed Model Under Different Attacks

**Figure 8:**
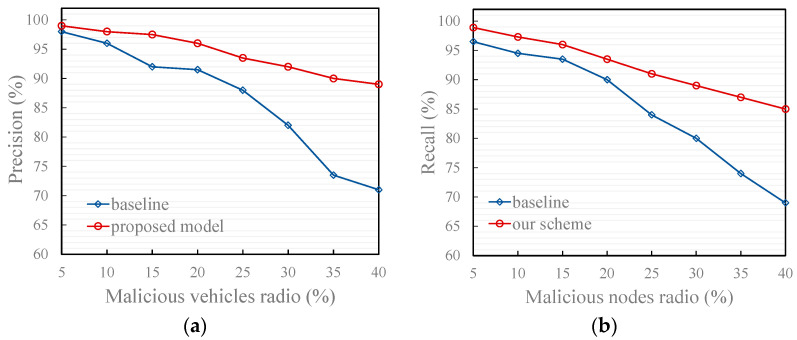
Effect of trust models in typical scenario 1. (**a**) Precision of the proposed and baseline model; (**b**) Recall of the proposed and baseline model. (The baseline in the figure refers to Reference [17]).

**Figure 9:**
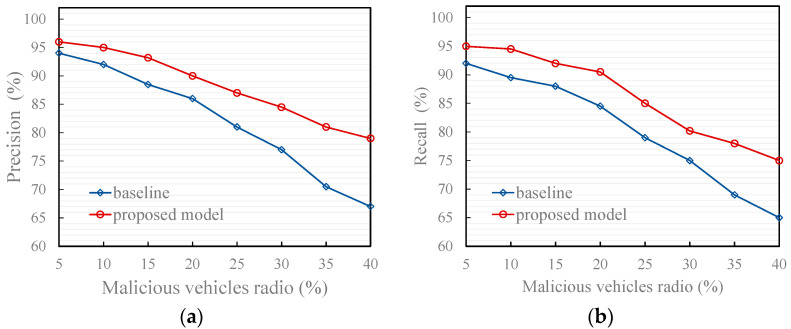
Effect of trust models in typical scenario 2. (**a**) Precision of the proposed and baseline model; (**b**) Recall of the proposed and baseline model.

**Figure 10:**
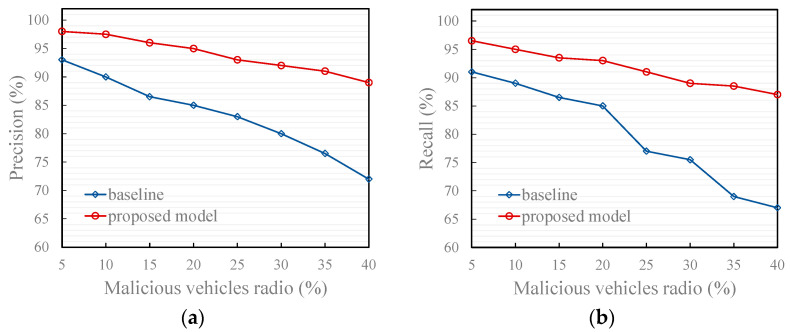
Effects of trust models under the attack model. (**a**) Precision of the proposed and baseline model; (**b**) Recall of the proposed and baseline model.

Figure 8, Figure 9 and Figure 10 illustrate the effectiveness of the proposed model compared to the recommendation-based model [17] in identifying the trustworthiness of traffic message under two typical scenarios and an attack model. The baseline refers to the results obtained by running the comparative model (the recommendation model from [17]) under the same simulation environment and data as our proposed model.

The recommendation-based trust model estimates each vehicle’s global trust by combining direct interactions with neighbor recommendations, and then determines the credibility of shared messages based on these global trust values. In contrast, the proposed truth-oriented trust model quantifies the integrated vehicle trust (I-VT) of each message sender by fusing self-experience-based vehicle trust (SEB-VT) and peer-recommendation-based vehicle trust (PRB-VT). Subsequently, using the I-VT values of participating vehicles, it computes the event trust (ET) by differentiating message attitudes and quantifying their relative influence, through which the trustworthiness of shared messages is finally evaluated. The critical difference lies in that the proposed model shifts the evaluation focus from individual vehicles’ historical trustworthiness to the truthfulness of shared events, enabling a message-centric, entropy-informed trust reasoning that captures both vehicle reliability and contextual message consistency.

As shown in the figures, both precision and recall decrease as the proportion of malicious vehicles (i.e., vehicles sending untrustworthy traffic message) increases. This decline is attributed to the growing frequency of false message injections, which increases the likelihood that such messages are misclassified as trustworthy. Moreover, a higher proportion of malicious vehicles amplifies the disturbance to trust evaluation, thereby weakening the accuracy of identifying genuine messages. Additionally, the proposed model outperforms the comparison model under all three attack models for the following reasons:

1. Typical scenario 1

As shown in Figure 8, under typical scenario 1 where malicious vehicles deliberately share false traffic messages that deviate from actual road conditions independently, the proposed model consistently outperforms the baseline models in both precision and recall. This superiority stems from its dual-layer defense design, which jointly enhances individual reliability assessment and group-level consensus validation.

First, the introduction of the smoothing factor *d*(*m*) in computing the self-experience-based vehicle trust (SEB-VT) effectively mitigates the instability caused by sparse behavioral evidence. In conventional trust models, a vehicle that has interacted only a few times may generate overconfident trust estimates, leading to a high risk of false positives when malicious messages appear. In contrast, our entropy-guided smoothing dynamically adjusts the trust update rate according to the uncertainty level—reducing the weight of unreliable small-sample observations while preserving responsiveness as evidence accumulates. This mechanism allows the model to maintain a cautious attitude toward vehicles with insufficient behavioral history, thereby lowering the probability of wrongly accepting false information.

Second, beyond individual evaluation, the model introduces a link analysis-based reference degree to quantify the credibility of recommendation information. Traditional trustor-centric approaches rely on a single evaluator’s perspective, making them vulnerable to localized cognitive bias. Our link analysis approach aggregates global endorsement of each recommender, forming a collective intelligence that reflects the overall reliability recognized by the vehicular network. This RSU-side computation captures both the quantity and quality of historical recommendations, enabling the system to down-weight isolated but untrustworthy recommenders whose messages are not broadly validated.

These findings substantiate that the combination of entropy-based smoothing and link analysis collectively provides both statistical and structural resilience against independent malicious behaviors, highlighting the model’s theoretical soundness and practical robustness in adversarial vehicular environments.

2. Typical scenario 2

Figure 9 illustrates that under typical scenario 2, in which malicious vehicles collude to disseminate false traffic messages, the proposed model shows a moderate decline in precision and recall compared with typical scenario 1. This degradation is expected, since collusive behaviors amplify the consistency and perceived reliability of false information through repeated mutual reinforcement among malicious participants. Such reinforcement increases the apparent agreement within the network, misleading conventional trust models that rely solely on local evaluations or simple majority voting.

Nevertheless, even under this challenging coordinated-attack scenario, the proposed model still significantly outperforms the baseline methods in both precision and recall. This robustness arises from its event-trust-based fusion mechanism, which introduces two complementary layers of defense against collusion.

First, at the message-sender level, each message is weighted according to the dynamic trust value of its originating vehicle. Messages from unreliable or low-credibility senders receive proportionally lower weights, effectively suppressing their collective influence even when malicious nodes attempt to reinforce each other. This design prevents the “echo-chamber” effect common in collusive environments, where mutual endorsements between attackers can rapidly inflate false credibility.

Second, at the message-aggregation level, the system distinguishes between positive and negative message sets according to their attitudes toward the same event. By processing these sets independently and aggregating their trust values separately, the model avoids the dominance of any single biased group of messages. This dual-set mechanism ensures that contradictory information can coexist and be assessed objectively, reducing the likelihood that collusive false messages overwhelm genuine evidence.

Compared with the baselines—which typically perform direct averaging or rely on unweighted majority fusion—the proposed approach explicitly models the provenance and inter-event diversity of information. This design grants the model a structural advantage: it not only mitigates the amplification effect of collusion but also preserves sensitivity to legitimate message diversity. Consequently, the proposed framework maintains a stable balance between false-message suppression and true-message retention, thereby demonstrating its adaptability and resilience under coordinated misinformation attacks.

3. Under the non-friendly recommendation attack mode

**Figure 11:**
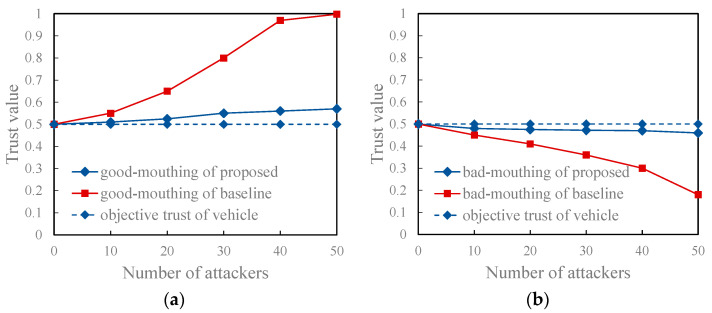
Trust values of regular vehicles under the attack model. (**a**) With different scales of good-mouthing attacks, proposed model vs. the baseline model; (**b**) With different scales of bad-mouthing attacks, proposed model vs. the baseline model.

As presented in Figure 10, under the non-friendly recommendation attack model, the proposed model shows excellent performance. This is because the proposed model leverages collective intelligence to compute the reference degree of recommended information. This computation is based on the quality and quantity of vehicles that have referenced the recommender’s historical recommendation information. Compared with the comparison method that only computes the reference degree through the subjective trust value of the trustor vehicle on the recommender, this method makes more reasonable use of each recommendation information and greatly reduces the possibility of malicious recommenders misleading evaluators. Additionally, Figure 11 shows that the trust value computed using the proposed model for a regular vehicle aligns more closely with the vehicle’s objective trust value, regardless of whether it is subject to a good-mouthing or bad-mouthing attack.

#### 4.3.2. Effects of the Proposed Model on the Driving Distance of AVs After Path Planning

**Figure 12:**
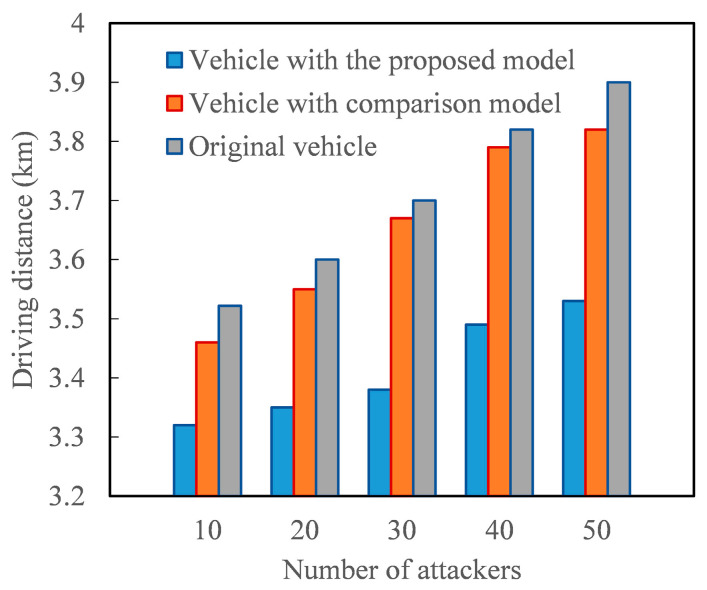
The driving distance of vehicle under different number of attackers.

Figure 12 demonstrates the positive impact of the proposed model on the driving distance of AVs after path planning. Path planning for AVs is an intelligent decision-making process that determines a vehicle’s best route based on the starting point, destination, and real-time traffic conditions. When the perception of real-time traffic conditions is mainly derived from message traffic message, the driving distance traveled by AVs increases with the number of malicious vehicles sharing false traffic message. This is because the increase in false traffic message raises the probability of AVs being misled and choosing detours. However, since the proposed model can reasonably identify the trustworthiness of message traffic message, deploying it in the path planning of AVs effectively reduces the impact of false traffic message on vehicle driving distance. For instance, consider a trip with a minimum travel distance of 3.2 km. When the number of malicious vehicles sharing fake traffic message is 50, the vehicles under the comparison model cover nearly 10% more distance compared to vehicles under the proposed model. In other words, the trustworthy evaluation model of message traffic message proposed in this study can enhance the driving efficiency of AVs.

## 5. Conclusions and Future Works

To ensure reliable traffic message sharing in the context of leveraging the IoV to address issues in current transportation systems, we have introduced a trust evaluation model that resists personal cognitive biases and assesses the trustworthiness of shared message about road events. First of all, we leverage link analysis techniques to compute the reference degree of recommendation information. Subsequently, we compute the peer-recommendation-based vehicle trust based on the recommendation information and their reference degree, and then combine the self-experience-based vehicle trust to obtain the integrated trust value of the connected vehicles. Finally, we incorporate the trust value of each collaborating vehicle to comprehensively compute the trustworthiness of the current event, thereby determining whether the shared traffic message is trustworthy. By incorporating entropy as a supplementary metric, we observed that the entropy of SEB-VT diminishes with larger sample sizes, thereby reinforcing the soundness of the proposed smoothing mechanism. Furthermore, our simulation results clearly indicate that, in comparison to the baseline model (proposed in [17]), our proposed model can more accurately pinpoint untrustworthy shared traffic message.

In future research, the proposed trust framework can be further extended to align with emerging paradigms in decentralized and intelligent vehicular networks. For instance, integrating blockchain-based federated learning mechanisms can enhance fairness and transparency in collaborative trust evaluation, as demonstrated by Chen et al. [25]. Moreover, with the rise of AI-defined vehicles that continuously fine-tune heterogeneous models over space and time [26], incorporating adaptive and context-aware trust calibration strategies may help maintain security, interpretability, and consistency in dynamically evolving environments.

## Figures and Tables

**Table 1:** Simulation Parameters.

Parameter	Value
Traffic scene range	4000 m × 4000 m
Number of roads	14
Vehicle distribution	Random
Total number of vehicles	100
*σ*	24
*A*_0_	0.5
*c*	0.85
α	0.5
β	0.5
Simulation time	300 iterations

**Table 2:** Attack Models.

Modes	Behavior of Vehicles
Typical scenario 1=	Vehicles deliberately share false traffic message that deviates from actual road conditions independently.
Typical scenario 2	Colluding with other vehicles to collectively share false traffic message that contradicts actual road conditions.
Attack model (non-friendly attack)	Recommenders excessively praise or discredit the collaborative vehicles.

## Data Availability

Dataset available on request from the authors.
